# Risk stratification with Breast Cancer Index for late distant recurrence in patients with clinically low-risk (T1N0) estrogen receptor-positive breast cancer

**DOI:** 10.1038/s41523-017-0037-3

**Published:** 2017-08-03

**Authors:** Brock Schroeder, Yi Zhang, Olle Stål, Tommy Fornander, Adam Brufsky, Dennis C. Sgroi, Catherine A. Schnabel

**Affiliations:** 1 Biotheranostics, Inc., San Diego, CA USA; 2 Linköping University, Linköping, Sweden; 3 Karolinska Institute, Stockholm, Sweden; 4 University of Pittsburgh School of Medicine, Pittsburgh, PA USA; 5 Massachusetts General Hospital, Boston, MA USA

## Abstract

Patients with early-stage, hormone receptor–positive breast cancer with favorable clinicopathologic features are often not recommended for extended endocrine therapy. However, even patients with T1N0 disease remain at significant risk of distant recurrence up to 15 years following 5 years of endocrine therapy, highlighting the need for further stratification based on individualized risk to select patients for extended endocrine therapy. In this study, the incremental utility of genomic classification to stratify clinically low-risk patients for late distant recurrence was evaluated using the Breast Cancer Index. In 547 T1N0 patients from two cohorts that were disease-free at 5 years post-diagnosis, Breast Cancer Index categorized 32 and 36% from each cohort, respectively, with high risk of late distant recurrence that was associated with significantly reduced distant recurrence-free survival (86.7 and 89.6%) between years 5–15 and 5–10 compared to Breast Cancer Index low risk (95.4%; *P* = 0.0263 and 98.4%; *P* = 0.008). Findings support consideration of genomic classification in clinically low-risk hormone receptor–positive patients to identify candidates for extended endocrine therapy.

## Correspondence/findings

Patients with early-stage, hormone receptor–positive breast cancer (HRBC) are at long-term risk for recurrence following 5 years of endocrine therapy.^1, 2^ A number of clinical trials have demonstrated that continuing adjuvant endocrine treatment beyond 5 years (extended endocrine therapy (EET)) in this population results in a statistically significant reduction in disease recurrence; however, the absolute benefit is modest (3–5%) and prolonged endocrine therapy is associated with adverse effects and risk of several serious toxicities. As a result, EET is often not recommended for patients with favorable clinical and pathologic prognostic features (e.g., node negative (N0), ≤2 cm (T1); lower grade).^3^ A recent EBCTCG meta-analysis of 46,138 patients investigated the risk of late distant recurrence (DR) in patients treated with 5 years of endocrine therapy based on clinicopathologic risk factors.^4^ In this study, patients with T1N0 disease had 4, 9, and 14% risk of DR at years 5–10, 5–15, and 5–20, respectively. Although patients with T1N0 disease are generally considered a clinically low-risk population, further stratification of risk for late DR and likelihood of benefiting from EET would better facilitate individualized treatment planning.

Breast Cancer Index (BCI) is a validated gene-expression based assay for patients with estrogen receptor–positive (ER+) early-stage breast cancer that reports both a prognostic risk assessment and an endocrine predictive component. The prognostic component of BCI is based on a gene-expression signature that was developed through the algorithmic combination of an endocrine response biomarker (*HOXB13:IL17BR* (H/I)) and a proliferation biomarker (Molecular Grade Index (MGI))^5, 6^ and has been validated in multiple randomized trial cohorts to significantly stratify patients for late DR.^6, 7^ The endocrine predictive component is based on H/I alone, wherein a high H/I ratio predicts likelihood of benefit from EET.^8^ The objective of the current study was to assess whether BCI significantly stratified patients with clinically low-risk disease based on risk of late DR.

Briefly, subset analyses of patients with T1N0 disease who were disease-free at 5 years post-diagnosis from two previously published independent validation cohorts of HRBC patients (Stockholm randomized trial cohort (*N* = 237) and a retrospective multi-institutional cohort (*N* = 210) treated at University of Pittsburgh Medical Center and Massachusetts General Hospital) were performed.^6^ The T1N0 subsets corresponded to 75% (*N* = 317) and 59% (*N* = 358) of the reported cohorts, respectively. Detailed assay methods and cohort characteristics were previously described.^6^ The investigation of tumor samples was approved by an Institutional Review Board at each institution; informed consent from patients was not required. Kaplan–Meier analysis was used to assess the risk of DR within BCI risk groups, and hazard ratios (HR), associated 95% confidence intervals (CI), and *p*-values were analyzed.

**Fig. 1:**
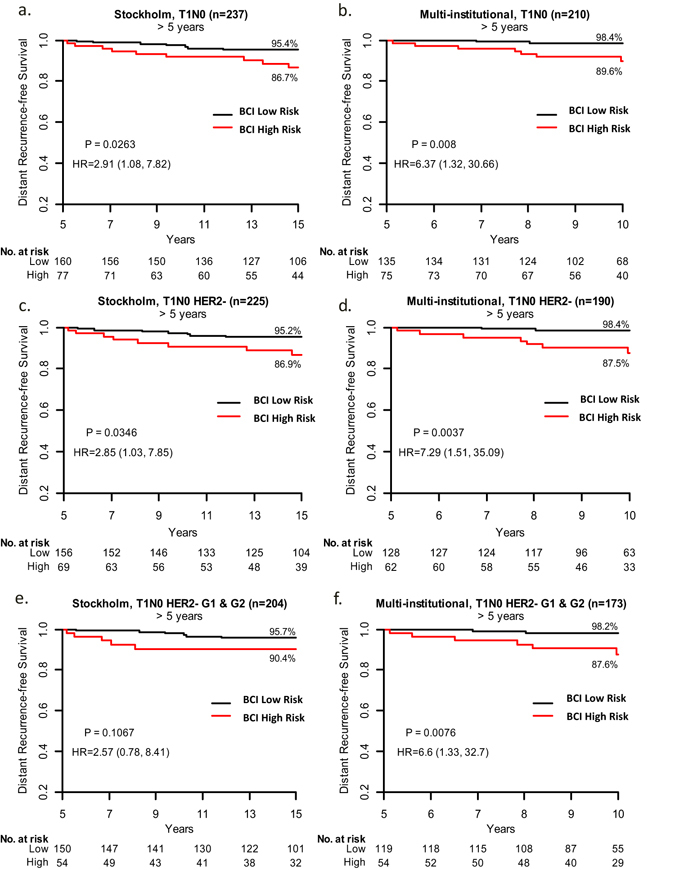
Kaplan-Meier analysis of prognostic performance of BCI in the Stockholm **a**, **c**, **e** and Multi-institutional **b**, **d**, **f** cohorts, respectively. **a**, **b** DRFS rates for T1N0 patients. **c**, **d** DRFS rates for T1N0, HER2^−^ patients. **e**, **f** DRFS for T1N0, HER2^−^, Grade 1 & Grade 2 patients. As described in previous study cohorts, BCI intermediate and high risk groups were combined for stratification of risk of late DR.

Patient baseline characteristics are summarized in Table 1. Median follow-up was 17 years and 10 years from diagnosis in the Stockholm and multi-institutional cohorts, respectively. In the Stockholm cohort, BCI identified 32% of T1N0 patients as high risk for late DR, and these patients had significantly lower DR-free survival (DRFS) between years 5–15 (86.7%) compared to BCI low-risk patients (95.4%; *P* = 0.0263; Fig. 1a). Similarly, in the Multi-institutional cohort, BCI identified 36% of T1N0 patients as high risk, and these patients had significantly lower DRFS between years 5–10 (89.6%) compared to BCI low risk (98.4%; *P* = 0.008; Fig. 1b). Within each cohort, 23 and 24% of patients classified as BCI high risk were T1a/b, and there was no significant interaction between BCI and T1 substage (T1a/b vs T1c; *p* = 0.19 and 0.74 for Stockholm and multi-institutional, respectively). Notably, within the BCI high-risk groups, 61 and 67% were classified as high H/I, respectively, indicating that the majority of these patients would be predicted to be likely to benefit from EET. Further subset analyses of patients with increasingly favorable clinical risk showed that BCI identified 31 and 33% of T1N0, HER2^−^ patients as high risk (Fig. 1c, d); and 26 and 31% of T1N0, HER2^−^, Grade 1/2 patients as high risk (Fig. 1e, f), respectively.

**Table 1:** Patient demographics and baseline characteristics for patients with T1N0 hormone receptor–positive breast cancer from the Stockholm randomized trial and multi-institutional cohorts

	Stockholm randomized trial cohort (*N* = 237)	Multi-institutional cohort (*N* = 210)
Age at surgery, y		
<50	2 (1%)	66 (31%)
50–59	68 (29%)	66 (31%)
60–69	156 (66%)	59 (28%)
≥70	11 (5%)	19 (9%)
Tumor size		
T1mi	0 (0%)	2 (1%)
T1a	7 (3%)	13 (6%)
T1b	76 (32%)	67 (32%)
T1c	154 (65%)	128 (61%)
Tumor grade		
Well	54 (23%)	63 (30%)
Moderate	158 (67%)	126 (60%)
Poor	25 (11%)	21 (10%)
PR status		
Negative	54 (23%)	NA
Positive	164 (69%)	NA
Unknown	19 (8%)	NA
HER2 status		
Negative	225 (95%)	190 (90%)
Positive	12 (5%)	20 (10%)
Received adjuvant chemotherapy		
No	237 (100%)	167 (80%)
Yes	0 (0%)	43 (20%)
Distant recurrences		
Late (>5 y)	16 (7%)	9 (4%)
*BCI risk group*		
Low	160 (68%)	135 (64%)
High	77 (32%)	75 (36%)
H/I category		
H/I Low	160 (68%)	123 (59%)
H/I High	77 (32%)	87 (41%)

NA, not available

Our findings suggest that molecular analysis of tumor biology can add resolution in assessing the risk of late DR for patients with clinically low-risk ER^+^ breast cancer. Across the 2 study cohorts, BCI identified a majority (64–68%) of T1N0 patients at low risk of DR, and these patients were associated with limited rates of late DR over the follow-up period, such that extension of endocrine therapy would unlikely result in further risk reduction. However, among this clinically low-risk population, BCI identified a notable subset of women as being at significantly higher genomic risk of late DR that would not have been identified using clinicopathologic evaluation alone, and would not generally be considered for EET. In addition, a majority of these patients were also classified as high H/I. Although this study aimed to evaluate the prognostic ability of BCI, a high H/I ratio significantly predicted benefit from endocrine therapy in two previous studies,^6, 8^ including in a cohort from the MA.17 study of patients randomized to extended letrozole or placebo following adjuvant tamoxifen.

The EBCTCG meta-analysis demonstrated that among patients with T1N0 disease who completed 5 years of endocrine therapy, the risk of late DR was 14% (~1% per year between years 5–20).^4^ However, despite the use of clinicopathologic factors, resolution at the individual patient level remains limited. Integration of a molecular assessment of tumor biology may enhance resolution. On the basis of the current BCI analysis, ~25% of T1N0 patients would be classified with high risk of late DR and predicted to benefit from EET based on a high H/I ratio. Although some degree of overtreatment continues to be a possibility, genomic classification can potentially improve the risk/benefit profile at the patient level. Notably, a small proportion of T1N0 (~12%) women were also classified as having a high risk of late DR but a low H/I ratio. Future studies should investigate additional regimens to reduce risk of recurrence (e.g., combinatorial approaches, CDK 4/6 inhibitors) in this patient subset. In patients with larger tumors (T2), a higher proportion were categorized as BCI high risk (53 and 55% in the Stockholm and multi-institutional cohorts, respectively) compared to T1, whereas the proportion of BCI high-risk patients with high H/I was similar in patients with T2 disease (67 and 65%, respectively).

Findings presented here demonstrate that BCI stratified clinically low-risk patients into genomically high-risk groups or low-risk groups with significant impact on outcomes. These results support potential use of genomic classification in patients with T1N0 disease to identify additional candidates for EET, and may be particularly relevant in younger patients who have a longer projected lifespan.

### Data availability

Supporting data can be made available upon written request for non-commercial purposes to researchers subject to a non-disclosure agreement with all relevant parties, and by contacting the corresponding author.
